# Supplementary material for: Network Bursts in 3D Neuron Clusters Cultured on Microcontact-Printed Substrates
Source: Micromachines (Basel). 2023 Aug 31;14(9):1703. doi: 10.3390/mi14091703 (PMC10534818; doi:10.3390/mi14091703)
Supplement: Supplementary file 1 [file micromachines-14-01703-s001.zip › Supplementary materials.pdf]

Network bursts in 3D neuron clusters cultured on microcontact-printed substrates

Qian Liang[1], Zhe Chen[2], Xie Chen[1], Qiang Huang[1], Tao Sun[1*]

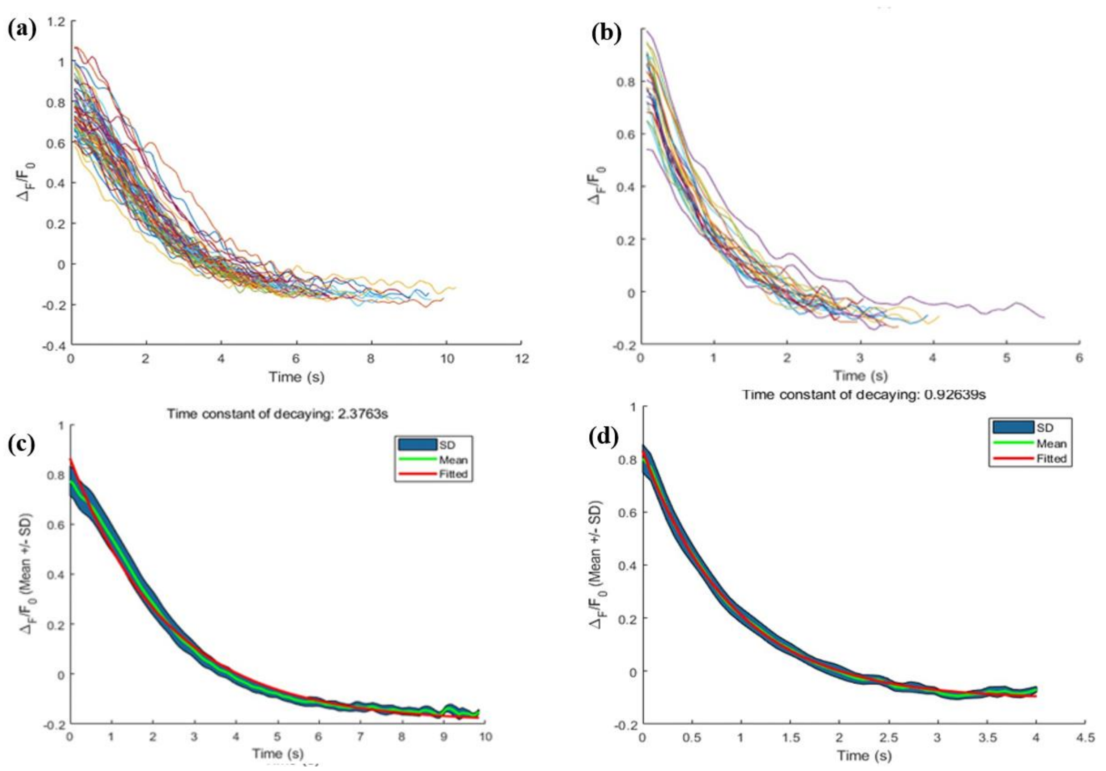

Figure. S1. Extracting and fitting the calcium decay phase to calculate the decay time constant. (a) and (b) Extracted traces of two neurons from day 15 and day 22, respectively. (c) and (d) small cluster large scale networks on show corresponding traces of mean and standard deviation (SD) belt, traces of fitted exponential function constant. The goodness of fit R^2 in (c) and (d) , and the decay time are 0.9909, and 0.9863, respectively. R^2 in fitting the time constant of decaying phase close to 1 on both day 15 and day 22 demonstrated the feasibility of the choice of exponentially decaying function and the good performance of the proposed method for extracting the decay phase. Data are presented with mean ±SEM.

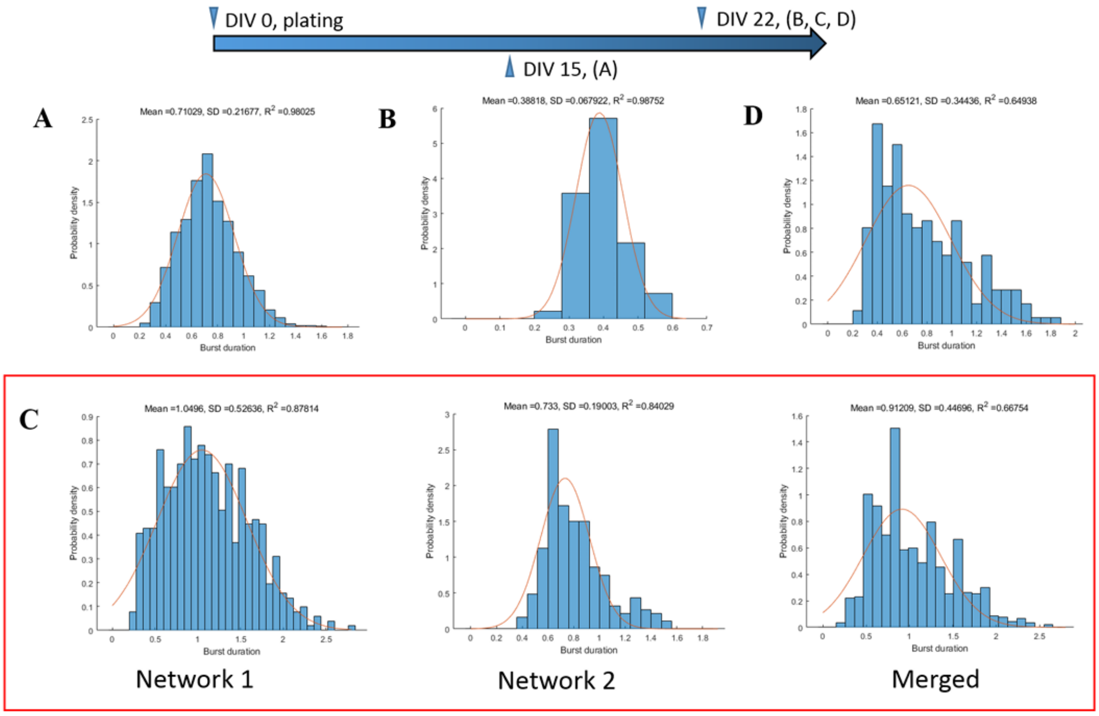

Figure. S2. The distribution of burst duration of different neuron networks. (A) 3D neuron cluster large-scale network (substrate 2) on day 15. (B) 3D neuron cluster large-scale network (substrate 2) on day 22. (C, left) Network 1, (C, middle) network 2, and (C, right) the merged one from two neighboring weakly separated networks with high clustering degree on day 22, as in Fig 5(a). (D) Another weakly separated network with low clustering degree on day 22, as in Fig. 5 (b). The high values of R2 in (A) and (B) showed that the burst duration in the small-cluster network was distributed normally. The difference of R2 in (C) indicated the burst duration of the two networks distributed normally but differently from each other. The low value of R2 in (D) suggested that the burst duration of this network was distributed non-normally. The orange curves represent the fitted Gaussian function, with the fitted mean, SD and R2 presented above each subfigure.

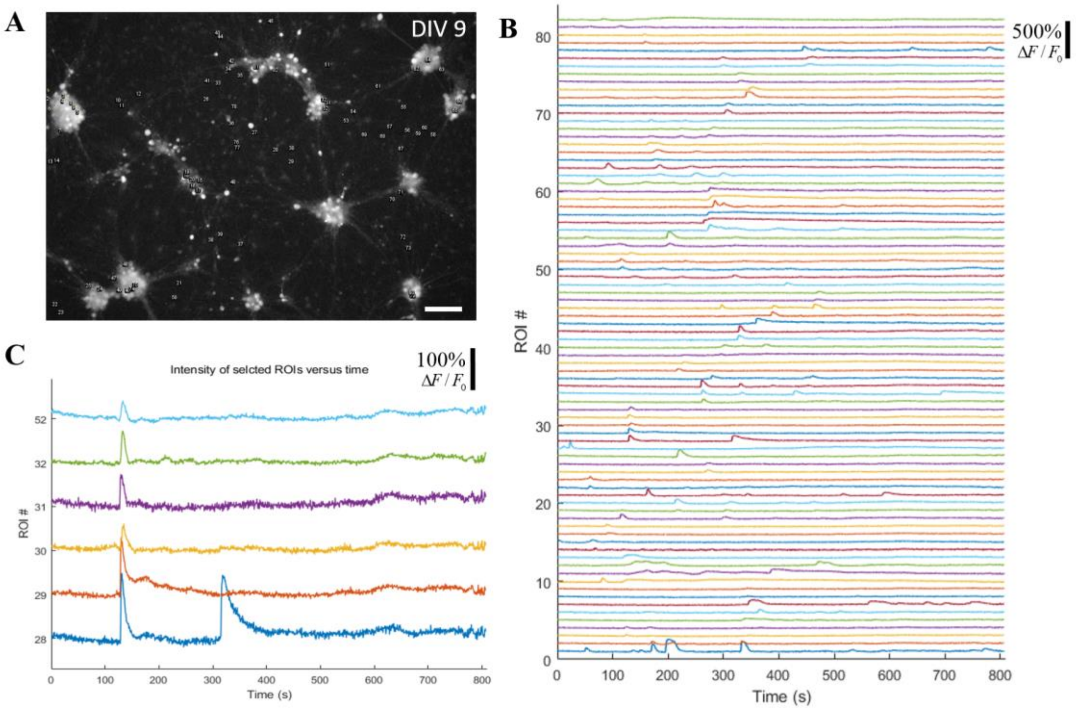

Figure. S3. ***Only subnetwork-wide burst activities were observed in the 3D neuronal network on DIV 9.*** *(**A**) ROI location in a small-cluster large-scale network on DIV 9. The grey image is the abstracted green channel of a peak-intensity frame of a calcium recording video. A total of 82 ROIs were selected manually. (**B**) Corresponding traces of relative fluorescence intensity of distinct ROI showed no network bursts, but only sporadic subnetwork bursts. (**C**) A six-neuron subnetwork synchronized calcium elevation on t = 140 s. Scale bar: 100 μm.*
